# Oocyte-granulosa cell interactions during mouse follicular development: regulation of kit ligand expression and its role in oocyte growth

**DOI:** 10.1186/1477-7827-4-19

**Published:** 2006-04-12

**Authors:** Fiona H Thomas, Barbara C Vanderhyden

**Affiliations:** 1 Department of Cellular and Molecular Medicine, University of Ottawa, and Centre for Cancer Therapeutics, Ottawa Health Research Institute, 501 Smyth Road, Ottawa, ON, K1H 8L6, Canada

## Abstract

Ovarian folliculogenesis is regulated by both endocrine and intraovarian mechanisms that coordinate the processes of oocyte growth and somatic cell proliferation and differentiation. Within the follicle, paracrine interactions between the oocyte and surrounding granulosa cells are critical for normal cell development and function. This review focuses on the role of paracrine interactions during early oocyte and follicular development that ensure proper coordination of oocyte and somatic cell function. Particular emphasis is given to granulosa cell-derived Kit Ligand (KitL), whose functional importance for oocyte growth has been demonstrated by a wide range of in vivo and in vitro studies. Reported interactions between KitL and oocyte-derived growth differentiation factor-9 (GDF9) and bone morphogenetic protein-15 (BMP15) suggest the molecular basis of oocyte-granulosa cell interactions, but also hint at the complexity of these communications. These paracrine interactions and the structure of the oocyte-granulosa cell interface are follicle stage-specific and regulated by FSH. Elucidation of the molecular mechanisms that promote the development of healthy oocytes with good developmental competence has potential applications for improving fertility and for in vitro growth systems for oocytes from domestic animals and humans.

## Introduction

Development of the ovarian follicle requires coordination of the processes of somatic cell proliferation and differentiation with oocyte growth and maturation [1]. Paracrine interactions between the oocyte and surrounding granulosa cells are critical for ensuring this coordination by promoting integrated cellular functions [2-4]. One of the first ligand-receptor systems to be identified in the ovarian follicle is Kit Ligand (KitL) and the receptor tyrosine kinase Kit. Since the identification of KitL in 1990 [5-7], numerous in vitro and in vivo studies have provided evidence to support its critical role in both female and male germ cell development. While there are several excellent review articles describing the importance of oocyte-granulosa cell interactions for normal follicular development [8-11], this review focuses on the particular role of the granulosa cell-derived KitL, and its interactions with the oocyte-specific growth differentiation factor-9 (GDF9) and bone morphogenetic protein-15 (BMP15/GDF9b). The role of FSH in regulating these paracrine interactions and the structure of the oocyte-granulosa cell interface during follicular development will also be addressed.

## Overview of follicular development

Ovarian follicles begin their development as primordial structures, which consist of an oocyte arrested at the diplotene stage of the first meiotic division, surrounded by a few flattened granulosa cells. In rodents, follicle formation occurs synchronously during the first few days after birth, whereas in domestic animals and primates, follicle formation occurs during fetal life (reviewed in [12]). In these species, follicles are formed over a much longer period and in a less synchronous manner. When follicles leave the resting pool, the oocyte grows and the granulosa cells proliferate to form a multi-laminar structure called a primary or preantral follicle. Once the follicle reaches a species-specific size, it forms a fluid-filled space called an antrum. When this stage has been reached, follicles become acutely dependent on gonadotropins for further growth and development. The growth phase of the oocyte occurs during the preantral stage in rodents, and it is during this time that development of the zona pellucida occurs, as well as production of mRNA and proteins required for subsequent fertilization and early embryonic development. These factors must be stored within the oocyte, as resumption of meiosis results in transcriptional silencing [13]. Oocyte developmental competence, defined as the ability of the oocyte to resume and complete meiosis, and support pre-implantation embryonic development after fertilization, is acquired gradually during folliculogenesis. Although acquisition of developmental competence is positively correlated with oocyte growth *in vivo*, the observation that oocytes cultured on fibroblasts do not grow, but can resume meiosis, indicates that these two events can be dissociated [14,15]. The dynamic changes associated with ovarian folliculogenesis are regulated by endocrine and intraovarian mechanisms which serve to promote and coordinate this growth [1].

## Paracrine control of oocyte-follicle interactions

Within the follicle, paracrine communications between the oocyte and surrounding granulosa cells are critical for normal cell development and function [8-11]. This review focuses on the role of paracrine interactions, in particular those involving KitL, during early oocyte and follicular development, and the effects of FSH on those interactions. In addition, the consequences of dynamic changes in the structure of the oocyte-granulosa cell interface during development will be addressed.

### Kit ligand-kit interactions

The tyrosine kinase receptor Kit and its ligand, KitL, have been localized to oocytes and granulosa cells, respectively [16]. KitL has been shown to stimulate mouse oocyte growth [17,18], and increased KitL in follicular fluid from women undergoing *in vitro *fertilization (IVF) has been correlated with successful pregnancies [19]. KitL is expressed in granulosa cells as either membrane-bound or soluble proteins arising from alternatively spliced mRNAs [20]. Soluble KitL (KitL-1) can be cleaved due to the presence of an 84 base pair exon (exon 6), which encodes a proteolytic cleavage site, allowing the extracellular domain to be released as a soluble product. Membrane-bound KitL (KitL-2) lacks this exon, is not efficiently cleaved and thus remains more stably on the membrane [20]. The ratio of *KitL-1/KitL-2 *mRNA differs between tissues [20], between ovaries of mice of different ages [21], and between granulosa cells of preovulatory and ovulatory rat follicles [22], suggesting that these transcripts are differentially regulated.

Kit, the receptor for KitL, is expressed in oocytes at all stages of follicular development in the mouse ovary [23], and several studies have demonstrated that female mice with naturally occurring mutations in *KitL *or *Kit *are infertile due to developmental abnormalities [23,24]. For example, mice homozygous for the *KitL*^*Sld *^allele, which produce only KitL-1, are sterile due to a deficiency in germ cells [25]. However, mice that exclusively produce KitL-2 are fertile [26], suggesting that KitL-2 may be the principal isoform required for oocyte development. Indeed, KitL-2 has been reported to induce a more persistent activation of Kit receptor kinase than the soluble form of KitL [27,28], and thus is likely to be the more potent isoform for regulation of oocyte growth.

In the two species (mouse and sheep) in which Kit/KitL and ovarian function have been most studied, there is evidence for expression of both ligand and receptor mRNA and/or protein, throughout the entire process of gonadal formation and follicular development, except in the early phase of meiosis I, prior to follicle formation [24,29,30]. In fetal gonads, an anti-apoptotic effect of Kit-KitL interactions on primordial germ cells, oogonia and oocytes has been demonstrated [24]. In postnatal ovaries, the initiation of follicular growth from the primordial pool and progression beyond the primary follicle stage appear to involve Kit-KitL interactions. Treatment of neonatal rat ovaries with recombinant KitL for 5 or 14 days *in vitro *increased the percentage of follicles in the growing pool, compared with ovaries cultured in control medium [31].

Yoshida *et al*. [32] have provided evidence of a role for Kit-KitL interactions at several stages of follicular development *in vivo*, by injection of an anti-Kit antibody into mice at different times during the first 2 weeks of life. A blockage of Kit function was found to affect the onset of primordial follicle development, primary follicle growth, follicular fluid formation, and preovulatory follicle development [32]. Moreover, it was shown that follicular growth was dependent on Kit during the first 5 days after birth, a period characterized by the absence of a functional FSH receptor [32]. These findings suggest a requirement for Kit signaling during FSH-independent follicular growth, as well as a role for Kit and its ligand in the maturing ovarian follicle.

In addition to its roles in the survival of fetal germ cells and initiation of follicular growth, there is increasing evidence for the importance of Kit/KitL activity for oocyte growth during preantral follicle development. Expression of both *KitL *and *Kit *in the ovary is consistent with a role for this ligand-receptor pair in oocyte growth [21], and a role for KitL in promoting early oocyte growth (in 8-day-old mice) *in vitro *has been demonstrated [17]. However, Cecconi *et al*. [33] reported no effect of soluble KitL on the growth of oocytes from 12-day-old mice, suggesting that KitL may have different actions at different stages of oocyte development. It may be that the actions of KitL are modulated by the presence of gap junctions, as Klinger and de Felici [34] have identified distinct stages of oocyte growth based on the ability of KitL alone to induce oocyte growth before transition to a growth phase that requires both KitL and contact with granulosa cells. Changes in the relative abundance of each KitL isoform may also permit differing actions at each stage of development. Work in our laboratory has shown that in oocyte-granulosa cell complexes (OGCs) cultured in the presence of a low concentration of FSH, the ratio of steady-state *KitL-1/KitL-2 *mRNA was decreased due to an increase in *KitL-2 *mRNA levels [18]. Importantly, this expression pattern was associated with increased oocyte growth in culture, suggesting that the correct balance of KitL-1/KitL-2 production is necessary for optimum oocyte growth *in vitro *[18]. In order to determine the specific role of each KitL isoform in promoting murine oocyte growth and maintenance of meiotic arrest *in vitro*, we have performed further experiments in which growing murine oocytes were cultured for 2 days on monolayers of KitL-deficient fibroblasts or on fibroblasts that stably express transfected constructs encoding either KitL-1 or KitL-2. The results of assessment of oocyte diameters and morphologies suggest that KitL-2 is the principal isoform required to promote the growth and survival of isolated growing oocytes [35].

### Kit signaling during follicular development

Binding of KitL to the Kit receptor leads to the phosphorylation of a set of cellular proteins via the kinase domain of the Kit receptor on its cytoplasmic tail [36-38]. Consequently, several signaling pathways that regulate apoptosis are activated via factors including Ras, Raf, mitogen activated protein kinase (MAPK) and protein kinase B (PKB/Akt) [39,40]. KitL stimulation also induces activation of phosphatidylinositol (PI) 3-kinase (PI3K), which is required for KitL-induced mitogenesis and survival [41]. In rat ovaries, Jin *et al*. [42] have demonstrated that one of the key downstream effectors of Kit activation is the PI3K/Akt module, through which the signal is transduced further downstream and converted into changes in expression of Bax and Bcl-xL that are important players in the apoptotic pathway. By using an inhibitor of PI3K during the culture of growing mouse oocytes on fibroblasts exclusively expressing KitL-2 (as described above), we have recently found that membrane-bound KitL-2 promotes the growth and survival of oocytes via a PI3K-mediated mechanism (Thomas and Vanderhyden, unpublished observations). Further experiments are being carried out to assess the levels of total Akt and activated Akt in oocytes cultured in the absence of KitL, and well as in the presence of KitL-1 or KitL-2.

### Growth Differentiation Factor-9 (GDF9) and Bone Morphogenetic Protein-15 (BMP15)

GDF9 has been shown to be expressed in several species, including human and mouse ovaries [43,44], and appears to be localized exclusively to oocytes at all stages of follicular growth, except primordial follicles, in neonatal and adult mice [44]. In *gdf9*^-/- ^mice, follicular development is arrested at the primary stage [45]. The pattern of *gdf9 *expression, as well as results from *gdf9 *gene knockout studies suggest that this factor may play an autocrine role in the regulation of oocyte development and maturation and/or function as a paracrine factor in the regulation of granulosa cell proliferation and differentiation [45,46]. BMP15 is an oocyte-specific homologue of GDF9, and has been cloned in mice [47]. In sheep, where this factor has been well studied, the Inverdale fecundity gene (FecX) carries an inactivating mutation in *bmp15 *[48], implicating this factor in the control of ovulation rate. As with GDF9, BMP15 has also been reported to play a role during several stages of follicular development [49].

In rodents, both GDF9 and BMP15 promote proliferation of granulosa cells from small antral follicles [50-52]. BMP15 has also been reported to inhibit FSH-stimulated progesterone production by rat granulosa cells [51], and is an inhibitor of luteinization [49]. At earlier stages of follicular development, *gdf9 *and *bmp15 *are predominantly expressed in oocytes of growing follicles; however, mRNA transcripts of these genes have been detected in primordial follicles of some species. In particular, in cattle, sheep and humans, expression of *gdf9 *was detected in oocytes of primordial follicles, whereas *bmp15 *expression was first detected in oocytes of primary follicles [48,53,43]. The significance of the species differences in the expression of these genes remains to be elucidated; however, a role for either GDF9 or BMP15 in activation of primordial follicles remains controversial. For example, ewes that were actively immunized against GDF9 became anovulatory and the ovaries contained few follicles beyond the primary stage [54], suggesting that the major role for GDF9 is from the primary stage onwards. Oocytes that were larger than those in normal primary follicles were also observed after immunization, reminiscent of those seen in *gdf9 *knockout mice. Immunization against BMP15 yielded a similar phenotype [54]. In contrast, recombinant GDF9 has been shown to promote the development of human primordial follicles to the secondary stage in culture, as well as improve follicular survival [55]. Following previous observations regarding the role of GDF9 in follicular development [45], new evidence has now emerged to suggest that GDF9 plays a role in the primordial to primary follicle transition in mice [56]. It is clear that more studies are required to determine the significance of GDF9 and BMP15 action in primordial follicles, as well as the role of these factors in follicular development in humans.

### GDF9 and BMP15 signaling

Members of the TGF-β superfamily signal via type-I and type-II receptors present on granulosa cells [49]. Upon ligand binding, the type-II receptors activate type-I receptors by phosphorylation; the receptors complex, then phosphorylate intracellular signaling molecules called Smads [57]. The specific Smad proteins whose activities depend on phosphorylation by type-I receptors are called receptor regulated Smads (R-Smads) [58-60]. The R-Smads includes Smads 1, 2, 3, 5 and 8. Once activated, the R-Smad molecules interact with another Smad, Smad 4, which is a common partner for all R-Smads. The Smad complex then translocates to the nucleus to interact with specific transcription factors to regulate the expression of target genes.

Although both GDF9 and BMP15 are members of the TGF-β superfamily, there is a distinct divergence of signaling pathways used by these oocyte-specific factors [57]. Both GDF9 and BMP15 signal through the BMP type-II receptor (BMPRII) [61]; however, GDF9 uses the ALK5 type-I receptor, and signals through the pathway activated by TGF-β and activin [62], whereas BMP15 signals through the ALK6 type-Ib receptor [63]. The intracellular signal for GDF9 is mediated through the phosphorylation of Smads 2 and 3, whereas the phosphorylation of Smads 1, 5 and/or 8 mediates the signaling of the majority of BMP ligands [49].

From examination of inherited patterns of ovulation rate in sheep, several breeds have been identified with point mutations in *bmp15*, *gdf9 *and *ALK6 *[64]. Five different point mutations have been identified in the *bmp15 *gene, one in *gdf9 *and one in *ALK6*. Animals homozygous for the *bmp15 *or *gdf9 *mutations are anovulatory whereas animals heterozygous for *bmp15 *or *gdf9 *mutations and those heterozygous or homozygous for *ALK6 *mutations have higher than normal ovulation rates. Moreover, immunisation of ewes against BMP15 or GDF9 shows that both are essential for normal follicular development and control of ovulation rate [65].

### Interactions between KitL, GDF9 and BMP15

**Figure 1:**
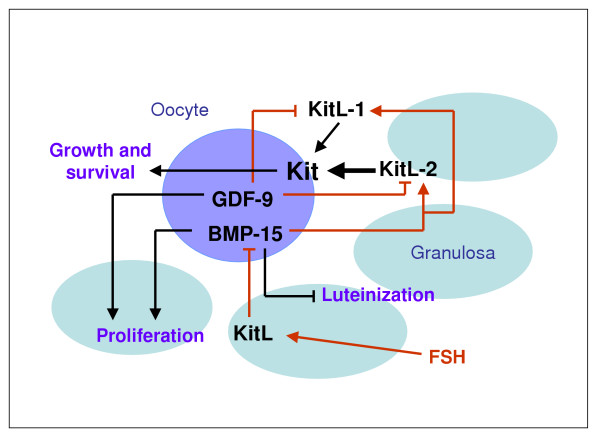
**GDF9, BMP15 and KitL interactions during oocyte and follicular development**. Interactions among factors derived from oocytes and granulosa cells are regulated by FSH, and are important for oocyte development. In rodents, both GDF9 and BMP15 have been shown to promote proliferation of granulosa cells from small antral follicles, and BMP15 has been reported to inhibit FSH-stimulated progesterone production, and is an inhibitor of luteinization. Evidence from studies with *gdf9*^-/- ^mice and granulosa cell cultures indicate that GDF9 suppresses expression of both *KitL-1 *and *KitL-2*. In contrast, BMP15 promotes *KitL *expression in monolayers of granulosa cells from rat early antral follicles and enhances the expression of both *KitL-1 *and *KitL-2 *mRNA in mouse oocyte-granulosa cell complexes grown *in vitro*. FSH is known to regulate *bmp15 *expression in a dose-dependent manner via Kit signaling, and Kit signaling promotes both oocyte growth and cell survival. Black lines indicate actions; red lines indicate effects on mRNA expression. The thick black arrow denotes the relative importance of KitL-2 in activating Kit receptors to promote oocyte growth.

While KitL, GDF9 and BMP15 may play distinct roles during follicular development, it is now clear that there are also significant interactions among these factors [18,66,67] (Figure 1). Recombinant GDF9 inhibits *KitL *mRNA expression in mouse preantral granulosa cells [66], whereas BMP15 promotes *KitL *expression in monolayers of granulosa cells from rat early antral follicles [67]. In addition, we have provided evidence of communication between BMP15 and KitL at the molecular level in intact murine OGCs *in vitro *[18]. By inhibition of Kit activity within OGCs *in vitro*, we have shown that FSH regulates *bmp15 *expression in a dose-dependent manner via Kit signaling [18]. Thus, interactions between oocyte- and granulosa cell-derived factors are regulated by FSH, which appears to fine-tune levels of expression of the paracrine factors.

Oocytes within *gdf9*-deficient mouse ovaries grow larger than normal [45,56]; however, these oocytes do not acquire full developmental competence [46]. Interestingly, these mutant mice also have elevated levels of *KitL-1 *and *KitL-2 *mRNA [46,56], which suggests that GDF9 regulates *KitL *expression. Differential regulation of the two *KitL *transcripts is likely to be a vital component in eliciting specific KitL-mediated actions during oocyte and follicular development. Although the mechanisms controlling the expression of the specific isoforms of KitL are not yet defined, it is likely cell-specific, as human chorionic gonadotropin has been shown to affect KitL isoform expression differently in mural vs. cumulus granulosa cells [22]. Stage-specific changes may also be important, since down-regulation of *KitL-2 *expression occurs during early antral follicle formation, coinciding with the cessation of oocyte growth [18]. Oocytes in *gdf9 *deficient mice may thus exceed the normal maximum diameter due to consistently elevated levels of *KitL-2 *expression.

## The role of FSH in regulating oocyte-follicle interactions

Endocrine control of follicular development by FSH rests on a network of intrafollicular paracrine interactions [68]. For example, FSH promotes proliferation and differentiation of preantral follicles via paracrine factors such as IGF-1 and activin [69,70]. In addition, FSH regulates *KitL *expression in granulosa cells from murine preantral follicles [71]. We have recently investigated the role of FSH in the regulation of *KitL *expression during the development of mouse preantral oocyte-granulosa cell complexes *in vitro *[18]. It was demonstrated that a low concentration of FSH decreased the ratio of steady-state *KitL-1/KitL-2 *mRNA by increasing *KitL-2 *mRNA levels, and this was associated with increased oocyte growth in culture [18]. These results suggest that the correct balance of KitL-1/KitL-2 production is necessary for optimum oocyte growth *in vitro*. In addition, the correct concentration of FSH is crucial for appropriate regulation of paracrine factors to promote oocyte development. In granulosa-luteal cells obtained after oocyte harvest from patients undergoing IVF, a decrease in *KitL *mRNA expression was reported in response to FSH and hCG in a time- and concentration-dependent manner *in vitro *[72], suggesting that KitL is hormonally regulated in humans and is likely to participate in follicular function during the human menstrual cycle.

Studies using mice with deficiencies in FSH receptor (FSH-R) expression have allowed further elucidation of the role of FSH in oocyte-granulosa cell interactions. It has been reported that mice lacking the FSH-R gene have structural alterations in the ovary at or before 2 days of age [73]. Furthermore, there is evidence to suggest that FSH-R deletion results in changes in oocyte structure and function, and disruption of oocyte-granulosa cell communication [74]. Specifically, oocyte growth was reduced in the FSH-R null mice, and the expression of Kit, KL and BMP15 proteins was decreased in both null animals and heterozygotes. These findings not only suggest that oocyte development is impaired when FSH signaling is impeded, but also that bidirectional communication between the oocyte and granulosa cells is influenced in a quantitative manner by FSH-R signaling events in the perinatal/postnatal period [74].

## Transzonal Processes (TZPs) and gap junctions mediate oocyte-granulosa interactions

Efficient delivery of factors to and from the oocyte at critical stages of development is essential for the coordination of oogenesis and folliculogenesis, and is influenced by the structural features of the oocyte-granulosa cell interface [75]. TZPs, which are granulosa cell extensions that traverse the zona pellucida and terminate on the oocyte cell surface, have been characterized by electron microscopy in many mammals, including mouse, rat and human [75,76]. These TZPs have been shown to undergo dynamic alterations in form and number during the course of follicular development [76]. They are most numerous at the preantral stage, forming adhesive and gap junctional contacts at the oolema. During peak periods of oocyte growth, TZPs extend as deep invaginations, impinging on the oocyte germinal vesicle [76]. FSH has recently been shown to regulate the ability of granulosa cells to make connections with the oocyte [77]. In that study, it was shown that FSH treatment of pre-pubertal or FSHβ-knockout mice decreased the density of TZPs, which coincided with changes in chromatin remodeling and acquisition of oocyte meiotic competence [77]. Clearly these processes are important for oocyte development, although the interactions between these structural features and the paracrine factors that control oocyte growth still need to be elucidated.

TZPs enable the formation of intercellular gap junctions that are necessary for normal follicular development. The stage-specific pattern of distribution of different types of the gap junctional proteins, connexins, has been established in murine follicles [78]. KitL has been shown to induce the onset of *in vitro *growth of isolated fetal mouse oocytes, in the absence of gap junctional communication with granulosa cells [34]. However, these oocytes were unable to progress to the final stages of growth, and there was a lack of synchrony between nuclear and cytoplasmic maturation. The authors noted that these oocytes had characteristics resembling oocytes from connexin-43 and -37-deficient mice, which have impaired follicular development beyond the preantral and early antral stages, respectively [79,80], thus it was hypothesized that the preantral/antral transition is a critical stage of oocyte development requiring the coordinated differentiation of the oocyte with the granulosa cells [34]. The maintenance of adequate communication between these two cell types during the preantral and early antral stages is therefore necessary to ensure subsequent oocyte developmental competence. Gittens *et al*. [81] have provided evidence for interplay between paracrine signaling and gap junctional communication. In that study, expression of *KitL*, *Kit *and *gdf9 *were analyzed in connexin-43 deficient mice, and the expression of connexin-43 was analyzed in *gdf9 *deficient mice. The results suggest that although gap junctional coupling among granulosa cells is not required to sustain expression of these paracrine factors, and GDF9 is not required to sustain gap junctional coupling among granulosa cells, granulosa cells must be coupled via connexin-43 gap junctions in order to optimally respond to GDF9 [81].

## Conclusion

At present, the major barrier to developing and optimizing *in vitro *techniques for alleviation of infertility in women is our lack of knowledge of how the oocyte acquires developmental competence during its growth within the follicle. There is substantial evidence to indicate that oocyte-granulosa cell interactions are essential for the proper coordination of oocyte and follicular development. Researchers during the past decade have developed and explored a variety of *in vitro *and *in vivo *model systems to reveal the identity and functions of several proteins, notably KitL, GFD9 and BMP15, which mediate at least some of the communication between oocytes and their surrounding granulosa cells. KitL is critical for the growth of oocytes, and its level of expression is differentially controlled by paracrine and hormonal factors. The early evidence suggests that gap junctional communication is not necessary to sustain KitL expression; however, both overall expression and the relative abundance of KitL isoforms are influenced by GDF9, BMP15 and FSH at various stages of follicular development. While current research suggests that KitL-2 is more efficient at stimulating oocyte growth, the molecular mechanisms that promote the acquisition of developmental competence remain unknown. Identification of the factors or patterns of expression that contribute to the growth of healthy oocytes with full developmental competence, and that can be used as indicators of oocyte quality, have potential impact on the establishment of *in vitro *growth systems for clinical application, as well as on pregnancy outcome and offspring health.
